# S100A11 functions as novel oncogene in glioblastoma via S100A11/ANXA2/NF‐κB positive feedback loop

**DOI:** 10.1111/jcmm.14574

**Published:** 2019-08-20

**Authors:** Yiming Tu, Peng Xie, Xiaoliu Du, Liang Fan, Zhongyuan Bao, Guangchi Sun, Pengzhan zhao, Honglu Chao, Chong Li, Ailiang Zeng, Minhong Pan, Jing Ji

**Affiliations:** ^1^ Department of Neurosurgery The First Affiliated Hospital of Nanjing Medical University Nanjing China; ^2^ Department of Neurosurgery The Affiliated Huai'an Hospital of Xuzhou Medical University, The Second People's Hospital of Huai'an Huai'an China; ^3^ Department of Pathology The First Affiliated Hospital of Nanjing Medical University Nanjing China; ^4^ Department of Neurology Brigham and Women's Hospital and Harvard Medical School Boston MA USA

**Keywords:** ANXA2, glioblastoma, NF‐κB, S100A11, ubiquitination

## Abstract

Glioblastoma (GBM) is the most universal type of primary brain malignant tumour, and the prognosis of patients with GBM is poor. S100A11 plays an essential role in tumour. However, the role and molecular mechanism of S100A11 in GBM are not clear. Here, we found that S100A11 was up‐regulated in GBM tissues and higher S100A11 expression indicated poor prognosis of GBM patients. Overexpression of S100A11 promoted GBM cell growth, epithelial‐mesenchymal transition (EMT), migration, invasion and generation of glioma stem cells (GSCs), whereas its knockdown inhibited these activities. More importantly, S100A11 interacted with ANXA2 and regulated NF‐κB signalling pathway through decreasing ubiquitination and degradation of ANXA2. Additionally, NF‐κB regulated S100A11 at transcriptional level as a positive feedback. We also demonstrated the S100A11 on tumour growth in GBM using an orthotopic tumour xenografting. These data demonstrate that S100A11/ANXA2/NF‐κB positive feedback loop in GBM cells that promote the progression of GBM.

## 1 INTRODUCTION

Glioblastoma (GBM) is a WHO grade IV of glioma.^1^, ^2^ Despite advances in surgery and chemoradiotherapy, the average survival time of GBM patients has changed little in recent years.^1^ It is therefore vital to elucidate the mechanisms of GBM development and identify molecular targets for this incurable cancer.

The S100 proteins are low molecular weight proteins (10‑12 kDa) that belong to the EF‑hand calcium‐binding proteins family and are only expressed in vertebrates.^3^, ^4^ S100A11(known as calgizzarin or S100C) is a member of the S100 proteins.^5^ S100A11 plays a different role in the growth regulation of human keratinocytes because it can both inhibit Ca^2+^‐induced growth and stimulate growth, by enhancing the activity of epidermal growth factor (EGF) proteins.^4^, ^6^ S100A11 is overexpressed in a number of cancers, such as papillary thyroid carcinoma, colon, pancreatic and ovarian cancers.^7^, ^8^, ^9^, ^10^ However, the role of S100A11 in GBM is not clear. Thus, this study was to analyse the function and mechanism of S100A11 in GBM.

Annexin A2 (ANXA2), belongs to annexin family, is a calcium‐dependent phospholipid‐binding protein. ANXA2 is overexpressed in numerous tumours, including glioma.^11^, ^12^, ^13^, ^14^ Aberrantly, expression of ANXA2 plays important roles in tumour development by regulating cell proliferation, apoptosis, invasion and metastasis.^15^

In this study, we investigated the roles of S100A11 in GBM tumorigenicity, particularly GBM cell proliferation, epithelial‐mesenchymal transition (EMT), migration, invasion and neurosphere formation via silencing or overexpression in GBM. We provide evidence that S100A11 promotes the development and progression of GBM via ANXA2‐mediated NF‐κB signalling pathway.

## 2 METHODS

### 2.1 GBM and nontumourous human brain tissues

The analysis of S100 family expression data was obtained from Cancer Genome Atlas (TCGA) database (http://tcga-data.nci.nih.gov). The correlation of S100 family expression with overall survival of GBM patients in TCGA was obtained from Gene Expression Profiling Interactive Analysis (http://gepia.cancer-pku.cn). Glioblastoma tissues and nontumourous brain tissues (NBTs) were provided by the First Affiliated Hospital of Nanjing Medical University. The NBTs were obtained from patients who underwent surgery after physical injury to the brain. This study was approved by the Ethics Committee of Nanjing Medical University. Written‐informed consent was obtained from each patient.

### 2.2 Cell culture

The GBM cell lines U251, U87, LN229, U118, A172 and T98 were bought from the Shanghai Cell Bank. Glioblastoma cells were cultured in Dulbecco's modified Eagle's medium (DMEM) with 10% foetal bovine serum (FBS). To induce glioma stem cells (GSCs) differentiation, GSCs were dissociated and cultured in Neurobasal media supplemented with N2, B27, 3 mM l‐glutamine and 5% FBS.

### 2.3 Lentivirus construction

For overexpression of S100A11 in U251 cells, the gene encoding human S100A11 was inserted into the pWPXLd‐puro plasmid for the expression of (Flag)‐S100A11 fusion protein. For silencing of S100A11 in U87 cells, short‐hairpin RNA (shRNA) was subcloned into the pLV‐puro plasmid. The sense target sequence for sh‐S100A11‐1 and sh‐S100A11‐2 were 5′‐GACAGAGUUCCUAAGCUUCTT‐3′ and 5′‐CUGGAAAGGAUGGUUAUAATT‐3′.

### 2.4 EdU assay

Cells were seeded in 96‐well plates and then stained in 50 μM EdU (5‐ethynyl‐20‐deoxyuridine, Ribobio) for 4 hour. The cells were fixed with 4% paraformaldehyde and permeabilizated with 0.5% Triton X‐100. After three washes with phosphate‐buffered saline (PBS), cells were reacted with 100 µL of 1 × Apollo^®^. The unclear was stained with DAPI and cells were photographed under fluorescence microscopy (Olympus).

### 2.5 Colony formation assay

Glioblastoma cells were seeded in 6‐well culture plates. After about 15 days, the cells were fixed in methanol for 20 min and stained 0.1% crystal violet solution. The plates were photographed by digital camera.

### 2.6 Transwell invasion assay

Cells were added to top of chambers precoated with Matrigel in serum‐free DMEM, and DMEM/F12 medium with 10% FBS was added to the lower chamber. After cultured for 36 hour, the invading cells were fixed with methanol and stained with a 0.3% crystal violet solution. Each well was randomly photographed by microscope.

### 2.7 Wound‐healing assay

Cells were cultured in 6‐well plates and grew to ~95% confluence. The linear wound was created by a plastic pipette tip. The linear wound was then photographed under a microscope at 0 or 48 hour.

### 2.8 3D migration assay

3D migration assay was performed as described previously.^16^

### 2.9 Flow cytometric assay

Flow cytometry assay was performed as described previously.^17^ Cell cycle distribution was determined using propidium iodide staining. The percentage of apoptotic cells was determined following Annexin V/PI staining.

### 2.10 Neurosphere formation assay

Neurosphere formation assay was performed as described previously.^1^ Extreme limiting dilution analysis was conducted using the software available at http://bioinf.wehi.edu.au/software/elda/.

### 2.11 Immunofluorescence and western blotting

Immunofluorescence and Western blotting were done as described previously.^18^ Antibodies against S100A11(ab180593), Cyclin D1(ab40754), Cyclin E1 (ab33911), Cleaved caspase‐3 (ab13585), Bcl‐2 (ab32124), Bax (ab32503), p‐IKKα (Thr23, ab38515) and Fibronectin (ab2413, Abcam); E‐cadherin (#3195), CD133 (#64326), Nestin (#33475), N‐cadherin (#13116), Nanog (#4903), Oct4 (#75463), Vimentin (#5741), ANXA2 (#8235), HA (#5017), Myc (#2276), Histone H3 (#4499), Actin (#3700), GAPDH (#5174), IκBα (#4814), IKKα (#11930), p65 (#8242) and p‐p65 (#3033, Cell Signaling Technology) were used for immunofluorescence and Western blotting.

### 2.12 Real‐time quantitative PCR

Real‐time quantitative PCR was done as described previously.^19^ GAPDH used as an internal control. Primers were as follows: S100A11: forward, forward, 5′‐GAGTCCCTGATTGCTGTCTTC‐3′ and reverse, 5′‐AGGGTCCTTCTGGTTCTTTG‐3.

### 2.13 Co‐immunoprecipitation assay

Co‐immunoprecipitation assay was done as described previously.^18^

### 2.14 Chromatin immunoprecipitation assay

Chromatin immunoprecipitation assay was done as described previously.^20^

### 2.15 Orthotopic tumour xenografting

Male BALB/c‐A nude mice (6 weeks old) were bought from Beijing Vital River Laboratory Animal Technology Co. Ltd. Sh‐S100A11 or shCtrl cells were harvested and resuspended in L‐15 medium. Tumours were generated by injection of 5 × 10^5^ transfected U87 cells.

### 2.16 Statistical analysis

The experiments were performed at least three times. Data were presented with mean ± SEM to Student's *t* test for pairwise comparison or ANOVA for multivariate analysis. *P* values < .05 were considered statistically significant.

## 3 RESULTS

### 3.1 S100A11 is up‐regulated in GBM patients and represents an independent prognostic factor

**Figure 1:**
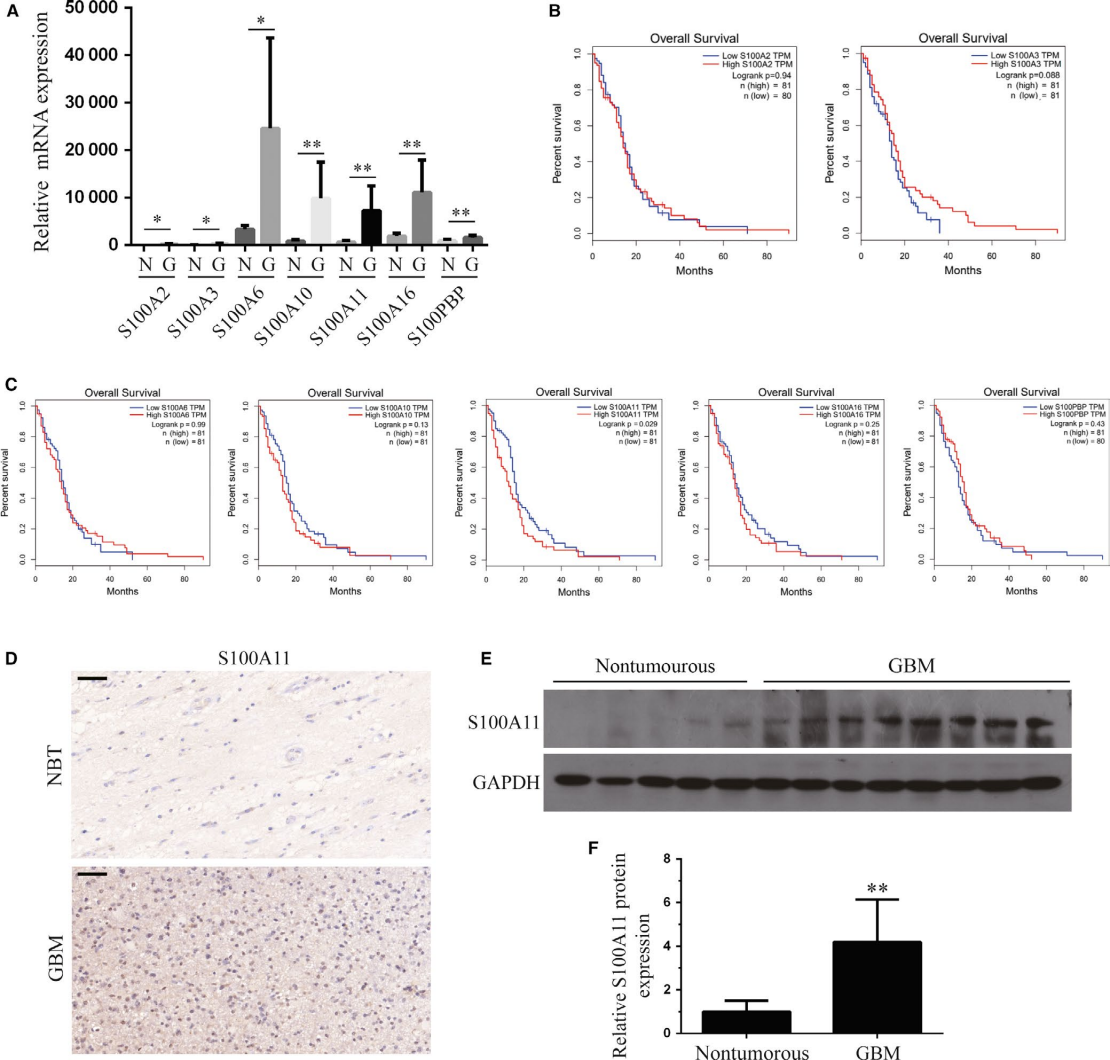
S100A11 is identified as oncogenic factor in GBM and predicts poor prognosis. A, Analyse the mRNA expression of S100 family members in GBM. S100A2, S100A3, S100A6, S100A10, S100A11, S100A16 and S100PBP were up‐regulated in GBM tissues compared with nontumourous brain tissues. G, GBM tissues; N, nontumourous brain tissues. B and C, Correlation of S100A2, S100A3, S100A6, S100A10, S100A11, S100A16 and S100PBP expression with overall survival for GBM patients. Only high S100A11 expression correlated with a poorer overall survival for GBM patients (*P < .05, **P < .01, log‐rank test). D, Representative images of IHC staining of S100A11 in nontumourous brain tissues and GBM tissues. Low expression of S100A11 was examined by IHC in a nontumourous brain tissues, while high expression of S100A11 was examined in GBM tissues, Scale bars, 50 μm. E and F, Western blot analysis of S00A11 expression in nontumourous brain tissues and GBM tissues (**P < .01). GAPDH was used as the loading control. Results are mean ± s.e.m. from at least three independent assays

Firstly, we examined the expression of S100 family in GBM from TCGA database, we found that S100A2, S100A3, S100A6, S100A10, S100A11, S100A16 and S100PBP were up‐regulated in GBM tissues compared with NBTs (Figure 1A). Then, by correlation of these up‐regulated S100 proteins expression with overall survival for GBM patients, we found that just high levels of S100A11 (*P* < .05) were a prognostic factor for poor overall survival in GBM patients (Figure 1B,C). In addition, we examined the expression of S100A11 in GBM by immunohistochemistry and Western blot. Immunohistochemistry was carried out in GBM tissues and NBTs slides (Figure 1D). The results of Western blot showed that S100A11 was up‐regulated in GBM samples compared with NBTs (Figure 1E,F).

### 3.2 S100A11 promotes cell proliferation of GBM

**Figure 2:**
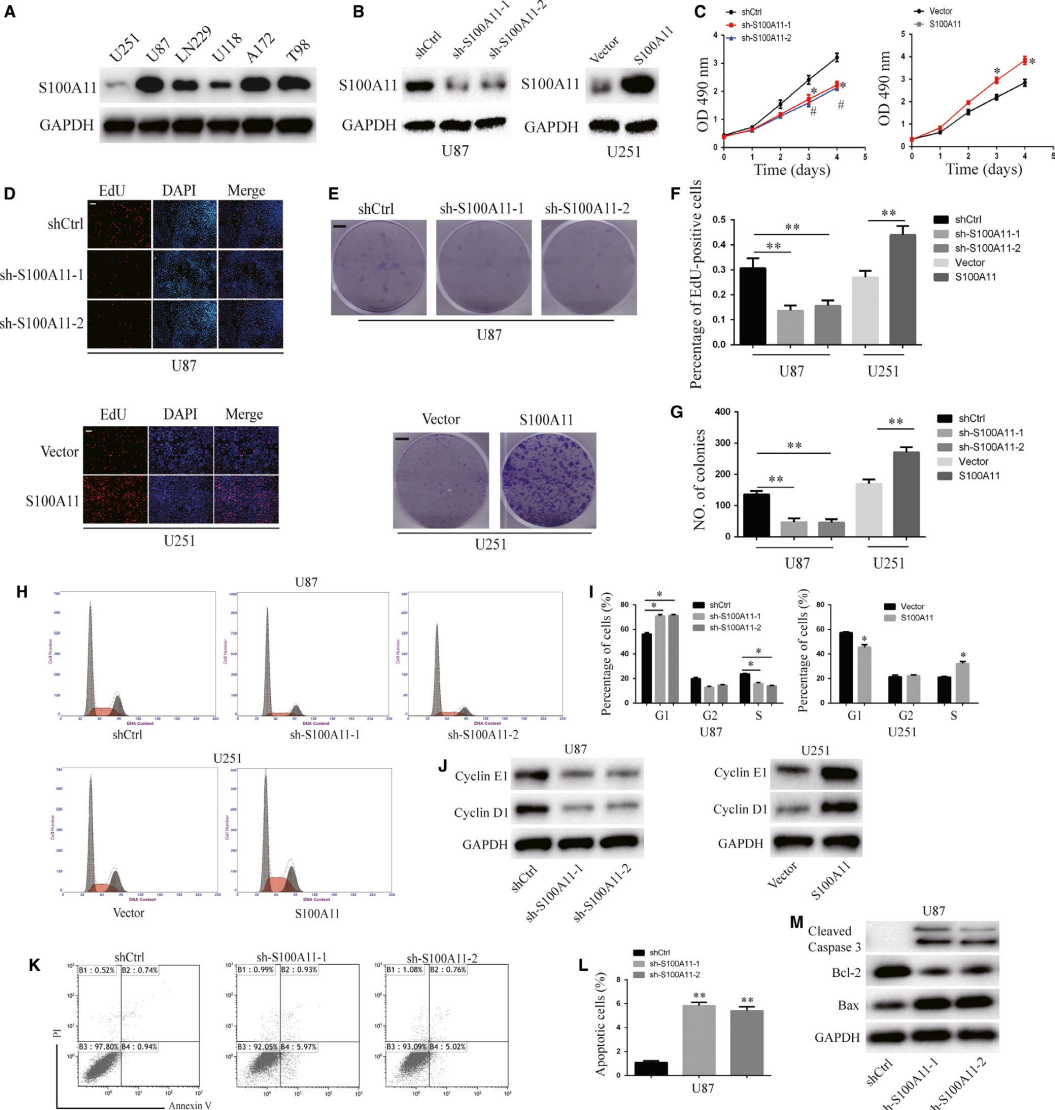
S100A11 induces the cell proliferation of GBM. A, Western blot analysis of S100A11 expression in six human GBM cell lines (U251, U87, LN229, U118, A172 and T98). B, Western blotting reveals that S100A11 was efficiently knocked down in U87 cell or overexpressed in U251 cells. C, CCK8 assay of the proliferation ability of the indicated GBM cells (*P < .05 and #*P* < .05). Results are mean ± sem. from at least three independent assays. D and F, Edu assay of the proliferation ability of the indicated GBM cells (**P < .01). Results are mean ± s.e.m. from at least three independent assays. Scale bar, 100 μm. E and G, Colony formation assay of the proliferation ability of the indicated GBM cells (**P < .01). Results are mean ± s.e.m. from at least 3 independent assays. Scale bar, 0.5 mm (H and I) Cell cycle assay of U87 cell after transfection with shCtrl, sh‐S100A11‐1 or sh‐S100A11‐2 and U251 cell transfection with Vector or S100A11(*P < .05). J, Western blot analysis indicated the regulation of the cell cycle regulatory proteins Cyclin E1 and Cyclin D1 in indicated GBM cells. GAPDH was used as the loading control. K and L, Annexin V/PI apoptosis assay of U87 cell after transfection with shCtrl, sh‐S100A11‐1 or sh‐S100A11‐2 (**P < .01). Results are mean ± sem. from at least 3 independent assays. (M) Western blot analysis indicated the regulation of the apoptosis‐related proteins Bax, Bcl‐2 and Cleaved Caspase‐3 in indicated GBM cells. GAPDH was used as the loading control

Then, we detected the expression of S100A11 in different GBM cell lines (U251, U87, LN229, U118, A172 and T98) by Western blot. S100A11 protein level was high in U87 cells and low in U251 cells (Figure 2A); therefore, we down‐regulated S100A11 in U87 and up‐regulated S100A11 in U251. The results of Western blot analysis with antibody against S100A11 confirmed the knockdown of S100A11 in U87 cells and overexpressing S100A11 in U251 cells (Figure 2B). CCK8, EdU and colony formation assays were used to detect the role of S100A11 in GBM cell proliferation. In CCK8 assay, downregulation of S100A11 significantly suppressed the proliferation of U87 cells, with its overexpression in U251 cells showing the opposite effects (Figure 2C). In EdU assay, downregulation of S100A11 significantly reduced the percentage of EdU‐positive U87 cells, with its overexpression in U251 cells showing the opposite effects (Figure 2D,F). Consistent with the above results, the results of the clonogenic assay revealed that S100A11 inhibition decreased colony formation and that overexpressing S100A11 increased colony formation (Figure 2E,G). Flow cytometric analysis showed that silencing of S100A11 arrested the GBM cells in the G1 phase (*P* < .01), whereas its overexpressing S100A11 resulted in more cells in the S phase and fewer cells in the G1 phase (*P* < .01, Figure 4H,I). Previous studies reported that Cyclin D1 and Cyclin E1 were related to the G1 arrest,^21^ we reported that knockdown of S100A11 decreased Cyclin D1 and Cyclin E1 expression levels and overexpression of S100A11 increased Cyclin D1 and Cyclin E1(Figure 2J). Annexin V/PI flow cytometric analysis showed that knockdown S100A11 increased in early‐phase apoptotic cells (Figure 2J,K,L). Knockdown S100A11 increased proapoptotic protein expression (Bax and Cleaved caspase‐3) and decreased the anti‐apoptotic protein expression (Bcl‐2) (Figure 2M). These results indicated that S100A11 promoted GBM cell proliferation.

### 3.3 S100A11 promotes cell EMT, migration and invasion of GBM

**Figure 3:**
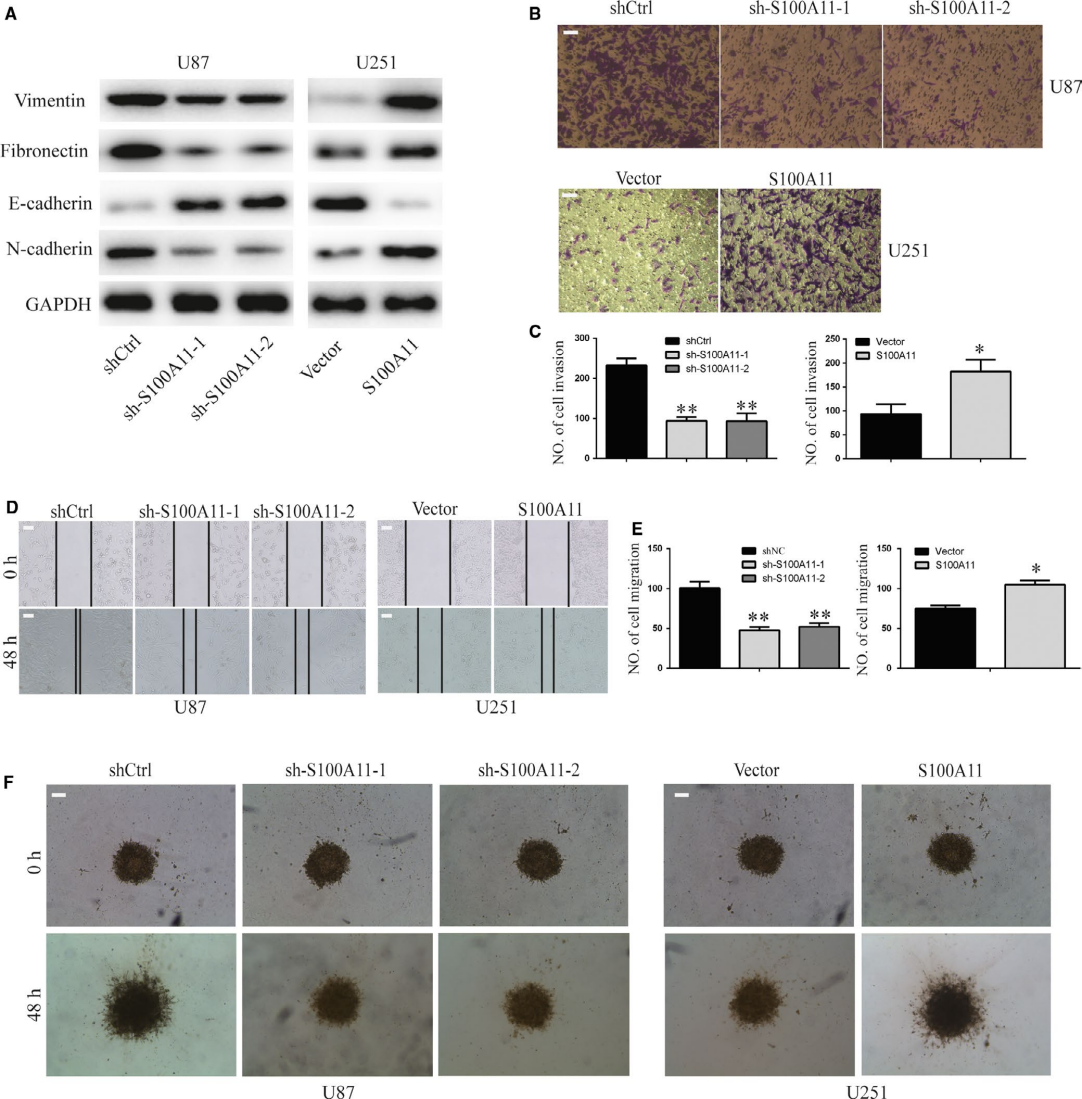
S100A11 promotes GBM EMT, migration and invasion. A, EMT‐associated proteins in indicated GBM cells were determined by Western blotting. GAPDH was used as the loading control. B and C, Transwell invasion assays of the invasive ability of the indicated GBM cells (*P < .05 and **P < .01). Results are mean ± s.e.m. from at least three independent assays. Scale bar, 100 μm. D and E, Wound‐healing assay of the migration ability of the indicated GBM cells (*P < .05 and **P < .01). Results are mean ± s.e.m. from at least 3 independent assays. Scale bar, 100 μm. F, 3D spheroid migration assay of the migration ability of the indicated GBM cells. Scale bar, 200 μm

Then, we determined the effect of S100A11 on EMT in GBM cells. The results showed that protein levels of vimentin, fibronectin and N‐cadherin were down‐regulated, while protein levels of E‐cadherin were up‐regulated in knockdown S100A11 U87 cells, with its overexpression in U251 cells showing the opposite effects (Figure 3A). Transwell system showed that downregulation of S100A11 in U87 cells reduced the number of invaded cells (*P* < .01). Overexpression of S100A11 in U251 cells yielded the opposite results (*P* < .05, Figure 3B,C). In the wound‐healing assay, the number of migrated cells at 48 hour was significantly decreased with S100A11‐silenced U87 cells (*P* < .01). Overexpression of S100A11 in U251 cells yielded the opposite results (*P* < .05, Figure 3D,E). Meanwhile, S100A11 downregulation inhibited cell infiltration in a 3D collagen matrix and S100A11 downregulating cells showed a less migratory morphology compared to that of the control cells. Overexpression of S100A11 in U251 cells yielded the opposite results (Figure 3F). Thus, S100A11 promotes GBM cell EMT, migration and invasion.

### 3.4 S100A11 promotes neurosphere formation

**Figure 4:**
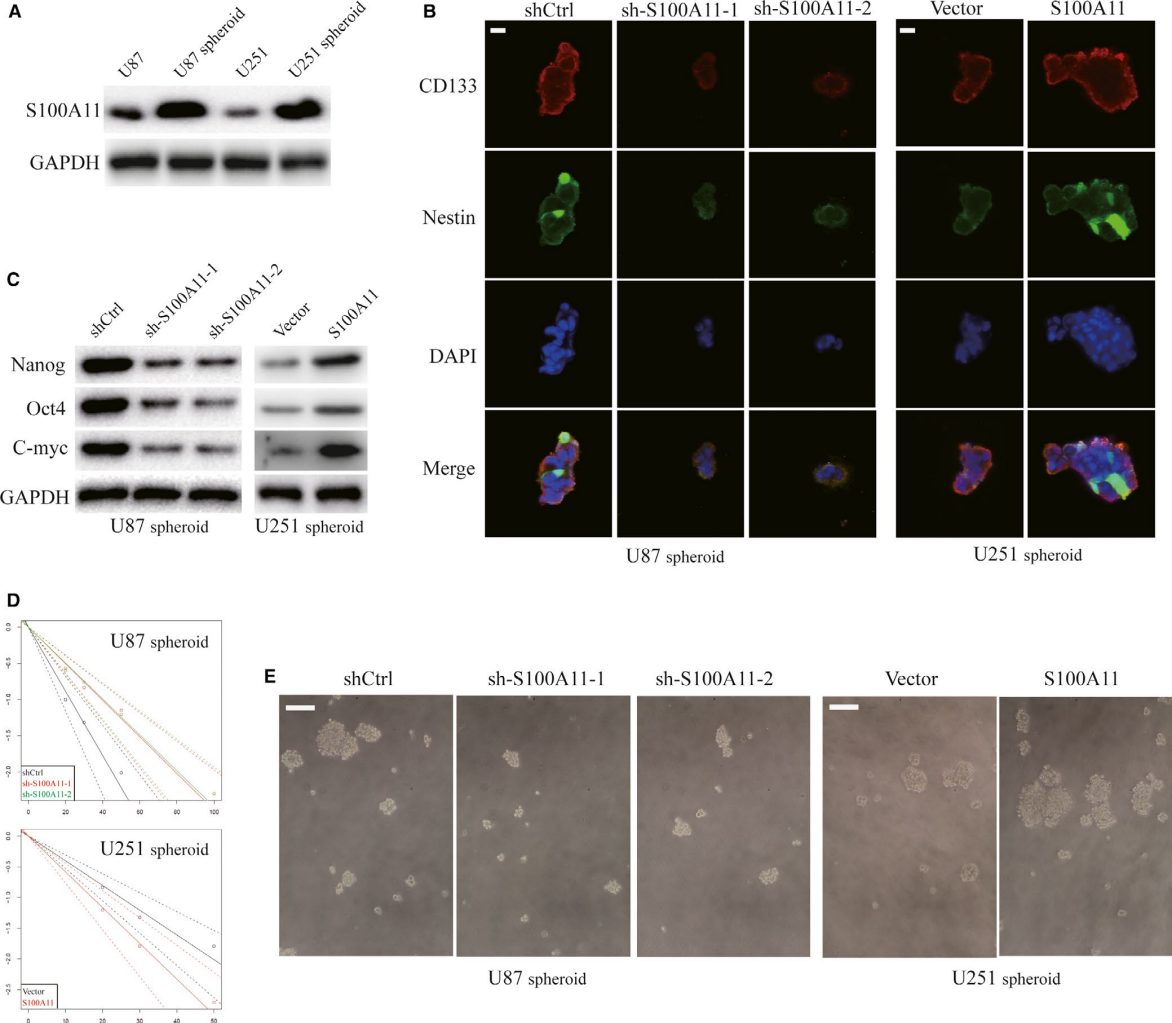
S100A11 facilitates the generation of GSCs. A, Western blot of S100A11 protein expression in neurospheres and adherent GBM cells. B, Representative immunofluorescence staining images of CD133 (red) and Nestin (green) in stem‐like U87 and U251 neurospheres. Scale bar, 20 μm. C, Western blot analysis of Sox2, Oct4 and Nanog in indicated GBM neurospheres. GAPDH was used as a loading control. D, Limiting dilution assay (LDA) was performed in indicated GBM spheres. E, Representative images of spheres formed in indicated GBM cells. Scale bar, 100 μm

We tested if S100A11 was associated with glioma stem cell (GSC). By suspension culture, U87 and U251 cell lines were employed to acquire neurospheres that might enrich GSCs. We observed that S100A11 protein level was significantly increased in neurospheres compare with that observed in adherent GBM cells (Figure 4A). Immunofluorescence staining indicated that the GSC markers (CD133 and Nestin) were expressed in U87 and U251 neurospheres (Figure 4B). Knockdown S100A11 markedly decreased GSC marker expression (Nanog, Oct4 and C‐myc). Meanwhile, overexpression of S100A11 yielded the opposite results (Figure 4C). By performing limiting dilution assays, we showed that S100A11 promoted neurosphere formation GBM cells (Figure 4D,E).

### 3.5 S100A11 stabilizes ANXA2 in GBM cells by decreasing ubiquitination and degradation

**Figure 5:**
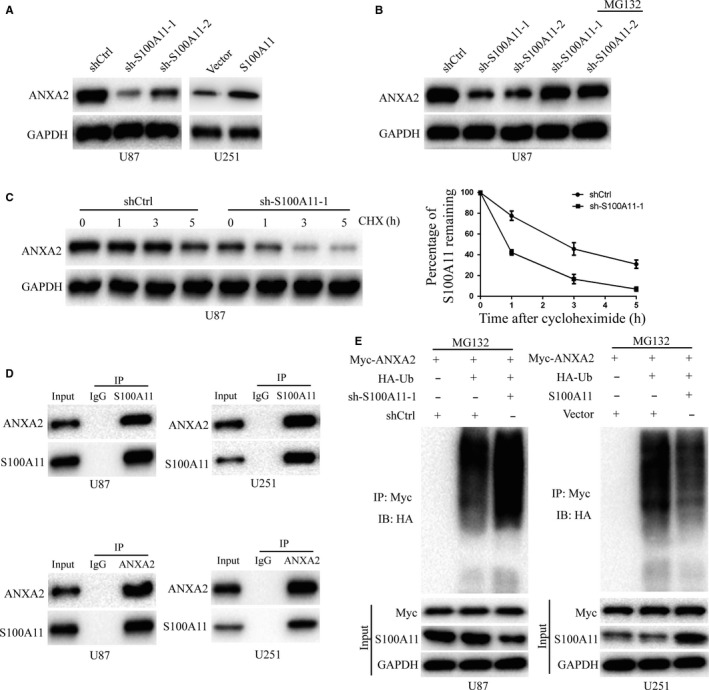
Proteasomal degradation of ANXA2 in the knockdown of S100A11. A, Knockdown of S100A11 decreased the expression of ANXA2, while overexpression of S100A11 elevated the expression of ANXA2. B, U87 transfected with shCtrl, sh‐S100A11‐1 or sh‐S100A11‐2, and the cells were treated with or without 10 μM of MG132, and the cell lysates were immunoblotted. C, U87 transfected with shCtrl, or sh‐S100A11‐1 were treated with 50 μg/mL cycloheximide. Whole‐cell lysates were harvested at the indicated times. Knockdown S100A11 decreased the half‐life of ANXA2. D, The interaction between S100A11 and ANXA2 was confirmed by co‐immunoprecipitation in U87 and U251 cells. E, U87 cells were co‐transfected with plasmids expressing HA‐Ub and Myc‐ANXA2 together with either shCtrl or sh‐S100A11‐1. U251 cells were co‐transfected with plasmids expressing HA‐Ub and Myc‐ANXA2 together with either Vector or S100A11 plasmid. Cells were treated with MG132 for 6 h before cell lysates were immunoprecipitated using a denature IP protocol to pull down ANXA2 protein, and the polyubiquitinated ANXA2 protein was detected by anti‐HA antibody

Next, we tried to search the mechanisms of S100A11 in GBM. Annexins and S100 proteins are two large but distinct calcium‐binding protein families.^22^ S100 proteins can interact with some annexins, such as ANXA1 and ANXA2.^22^ Ca^2+^ can trigger S100A11‐ANXA2 interaction.^22^ Additionally, ANXA2 promotes glioma cell invasion and tumour progression. For these reasons, we proposed that S100A11 promoted the development and progression of GBM may associate with ANXA2. Firstly, we examined the protein expression of ANXA2 in GBM cells and tissues by Western blot. The results showed that ANXA2 was up‐regulated in GBM cells compared with normal human astrocytes (NHAs) and up‐regulated in GBM tissues compared with nontumourous human brain tissues (Figure S1A,B). Then, we performed immunohistochemical staining of S100A11 and ANXA2 in twenty GBM specimens. As shown in Figure S1C, S100A11 is strongly correlated with ANXA2 expression. Then, Western blots showed that the protein level of ANXA2 was decreased when S100A11 inhibition, while ANXA2 was increased when S100A11 overexpression (Figure 5A). Previously, studies reported that reported ANXA2 degradation was regulated by poly/multi‐ubiquitin.^23^ We wondered whether knockdown of S100A11‐induced ANXA2 decreased through the ubiquitin–proteasome pathway. To detect the role of the proteasome in ANXA2 degradation, U87 cells were transfected with sh‐S100A11‐1, sh‐S100A11‐2 or shCtrl and treated with the proteasome inhibitor, MG132. The result demonstrated that ANXA2 was increased in knockdown S100A11 cells after treated with MG132 (Figure 5B). Moreover, the protein half‐life of ANXA2 was shortened after the knockdown S100A11 (Figure 5C). Then, the interaction between S100A11 and ANXA2 was using co‐ip in U87 and U251 cells (Figure 5D). More importantly, our findings showed that knockdown S100A11 increased the amount of polyubiquitinated Myc‐ANXA2 (Figure 5E). In contrast, the amount of ubiquitination of ANXA2 decreased in cells with up‐regulated S100A11 (Figure 5E).

### 3.6 S100A11 activates the NF‐κB pathway by ANXA2, and NF‐κB regulates S100A11 at transcriptional level

**Figure 6:**
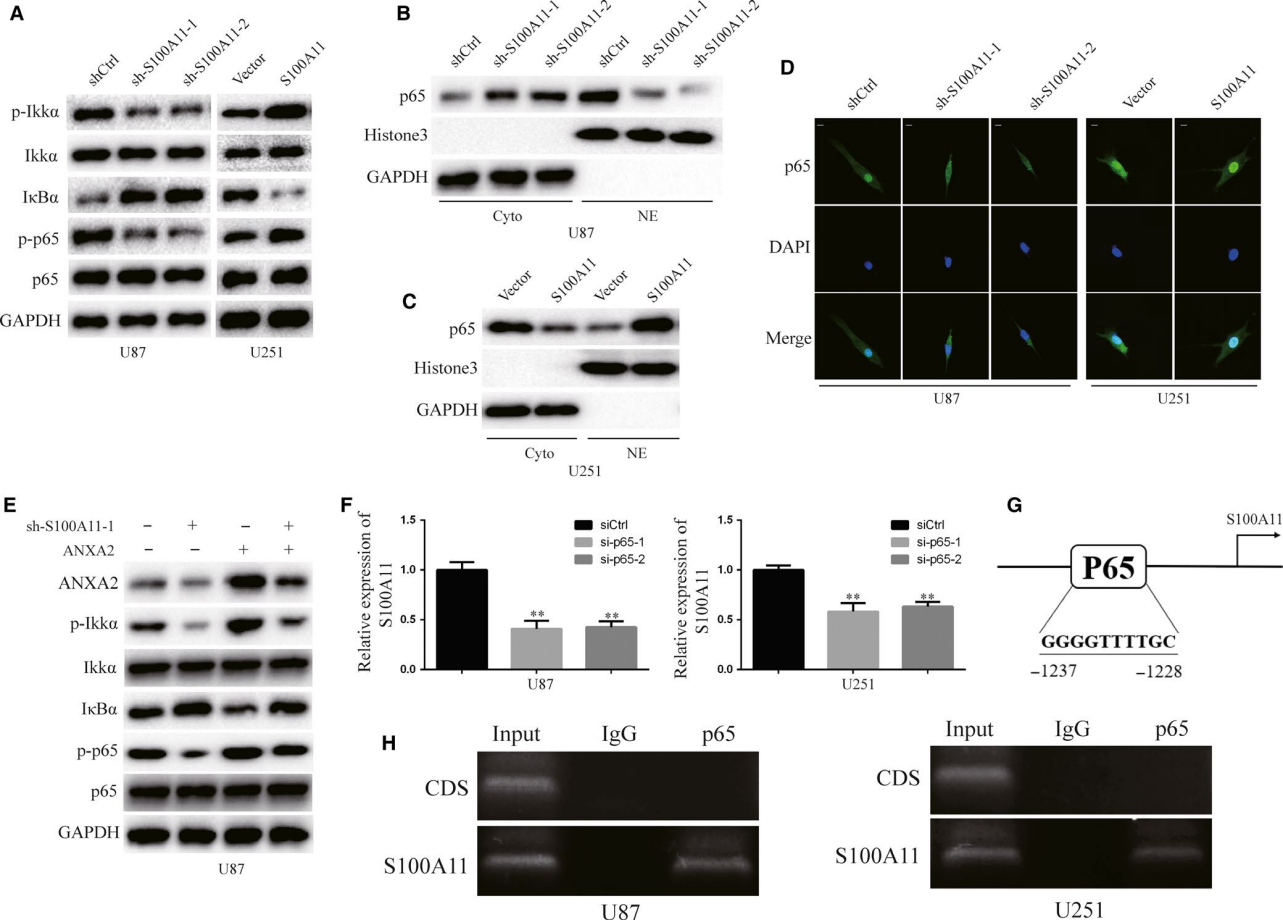
S100A11 activates NF‐κB pathway through ANXA2, and p65 regulates S100A11 at transcriptional level. A, Western blot analysis p‐IKKα, IKKα, IҡBα, p65 and p‐p65 in indicated GBM cells. GAPDH was used as the loading control. B and C, Western blot analysis of p65, GAPDH in cytoplasmic extracts (Cyto) and p65, Histone3 in nuclear extracts (NE) in indicated GBM cells. D, Immunofluorescence assay was conducted the distribution of p65 in indicated GBM cells. Scale bar, 20 μm. E, Exogenous expression of ANXA2 rescued the NF‐κB pathway inhibited by knockdown of S100A11. F, P65 was knockdown in U87 and U251 cells. S100A11 levels in U87 and U251 cells were measured by qRT‐PCR. G, A diagram shows a potential p65‐binding site in the promoter region of S100A11. H, Chromatin immunoprecipitation assays detected the binding of p65 with the S100A11 promoter in U87 and U251 cells. Normal rabbit IgG was used as a control. The resultant DNA was analysed with semiquantitative PCR using site‐specific primers

NF‐κB is closely associated with the progression of numerous cancers.^24^, ^25^ ANXA2 is closely associated with NF‐kB pathway.^11^, ^13^, ^26^, ^27^ Here, we detected whether the NF‐κB signalling was involved in S100A11/ANXA2 signalling in GBM. Firstly, we detected the total and phosphorylation levels of IKKα and p65, and total levels of IκBα expression in down‐regulated and up‐regulated S100A11 GBM cells (Figure 6A). The expression of p65 did not change in down‐regulated or up‐regulated S100A11 GBM cells compared to that in their control cells (Figure 6A). However, the results of Western blot and immunofluorescence demonstrated that p65 expression levels were induced in cytoplasmic and decreased in nuclear of knockdown S100A11 U87 cells compared to control cells. Overexpressing S100A11 yielded the opposite results (Figure 6B‐D). Additionally, overexpression of ANXA2 rescued NF‐κB activation that was inhibited by knockdown S100A11 (Figure 6E). Moreover, p65 is a classical transcription factor.^28^ Next, we determined whether p65 could regulate S100A11 at transcriptional level. P65 inhibition significantly decreased S100A11 mRNA levels in GBM cells (*P* < .01, Figure 6F). Scanning the S100A11 promoter region by JASPAR database (http://jaspar.binf.ku.dk/), we found one putative S100A11 binding site (Figure 6G). To examine the endogenous binding of p65 to the binding site in the promoter region, we performed ChIP assays and found that p65 did indeed bind to the site, but not to the coding region of S100A11 (Figure 6H). Therefore, our finding demonstrated that S100A11 activated the NF‐κB pathway by ANXA2, and NF‐κB regulated S100A11 at transcriptional level.

### 3.7 S100A11 promotes tumorigenicity in vivo

**Figure 7:**
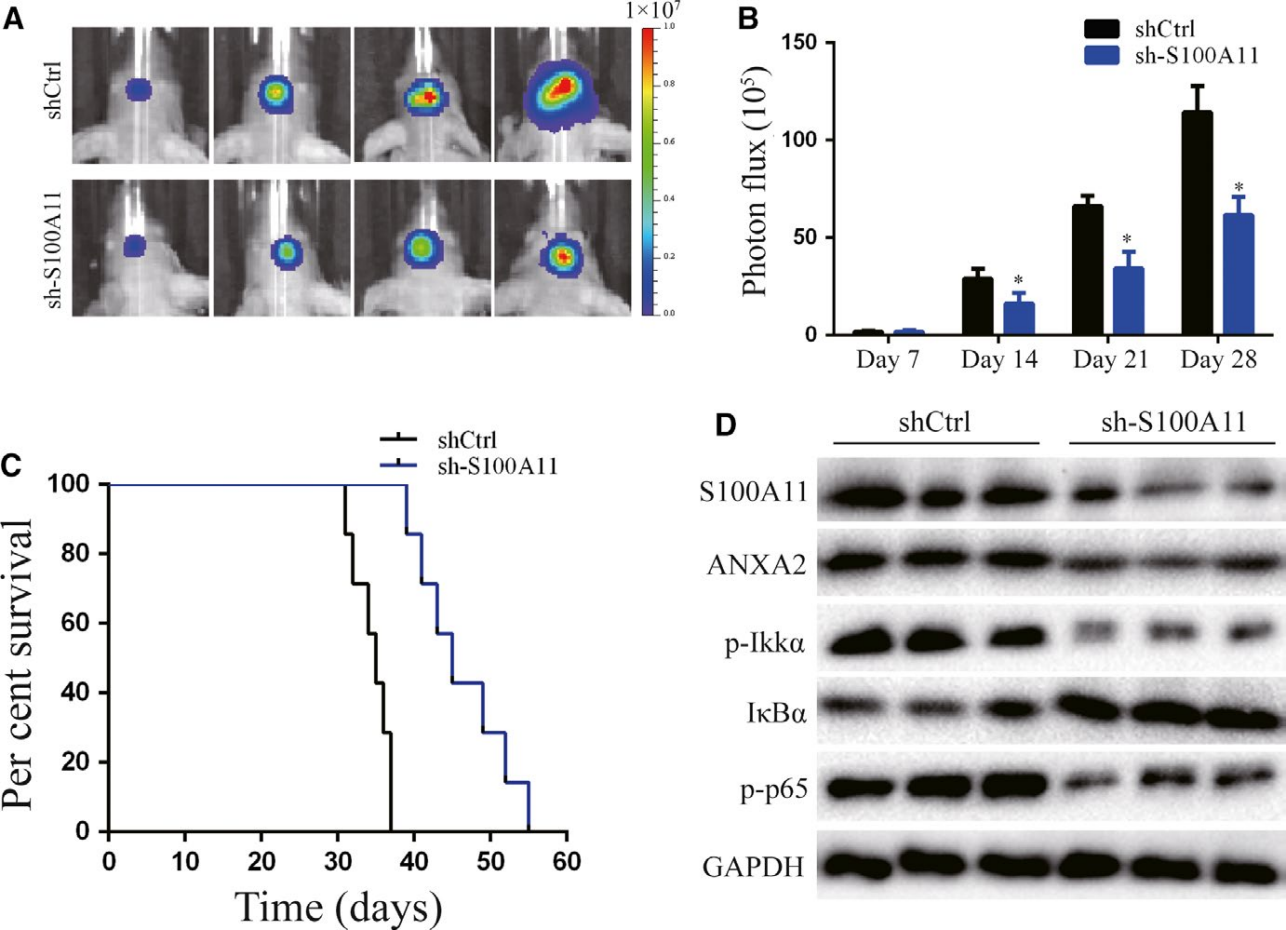
S100A11 promotes tumorigenicity in vivo. A and B, Bioluminescence images of orthotopic tumours bearing control or S100A11‐depleted U87 cells on the days as indicated. *P < .05. C, Survival curve of shCtrl‐transplanted or sh‐S100A11‐transplanted intracranial xenografts. D, Western blot analysis S100A11, ANXA2, p‐IKKα, IҡBα, and p‐p65 in U87 xenografts. GAPDH was used as the loading control

The effect of S100A11 in vivo was assessed using a U87 orthotopic xenograft model. As shown in Figure 7A,B, in vivo imaging system revealed that significant differences in the tumour volume between U87 cells transfected with sh‐S100A11 and with its control, mice bearing U87 cells transfected with sh‐S100A11 displayed a significant reduction compared with xenografts transfected with shCtrl. In survival curves, sh‐S100A11‐transplanted xenografts exhibited significantly increased survival as compared with shCtrl‐transplanted xenografts (Figure 7C). Moreover, important proteins, such as S100A11, ANXA2 and NF‐κB signalling proteins, were significantly inhibited by down‐regulated S100A11 in GBM xenografts (Figure 7D).

## 4 DISCUSSION

S100A11 is one of the S100 proteins that is widely expressed in human tissues. S100A11 has dual functions: it acts as tumour suppressor or tumour promoter on different tumour type. In keratinocytes, phosphorylation of S100A11 and the SMAD heterologous complex binds Sp1 proteins together in the nucleus.^29^, ^30^ The decrease in free Sp1 proteins induces p21 and inhibits DNA synthesis. Thus, under these conditions, S100A11 acts as a tumour‐suppressor factor. S100A11 can also stimulate the proliferation of human pancreatic cancer cells and promote the G1/S phase transition of pancreatic cancer cells, the growth and invasion of ovarian cancer cells, and the growth of lung cancer cells.^10^, ^31^, ^32^ However, S100A11 has not been reported in GBM, and the molecular mechanisms of S100A11 function in human GBM were unclear and required clarification.

In this study, we showed that S100A11 was up‐regulated in GBM and S100A11 was positively associated with poor patient survival. Functional studies demonstrated that S100A11 enhanced GBM cell proliferation, EMT, migration, invasion and neurosphere formation. Taken together, high expression of S100A11 is an important oncogenic factor and plays vital roles in the development and progression of GBM.

The molecular mechanisms of S100A11 by which promote the development and progression of GBM is unknown. ANXA2 is a calcium‐dependent phospholipid‐binding protein.^33^ ANXA2 is overexpressed in various tumours, including breast cancer, pancreatic cancer, ESCC and glioma, which plays important roles in tumour progression by regulating cell growth, apoptosis, invasion and tumour neovascularization.^14^ Calcium can trigger S100A11‐ANXA2 interaction in repair cell membrane.^22^ It is possible that S100A11 could interact with ANXA2 to promote the progression of GBM. This study, we showed that S100A11 could interact with ANXA2 and stabilize ANXA2 in GBM cells. Further mechanistic studies demonstrated that S100A11 could stabilize ANXA2 through decreasing ANXA2 ubiquitination and proteasomal degradation.

NF‐κB signalling is associated with numerous of cellular responses, such as cell survival, differentiation and proliferation.^25^ NF‐κB signalling has been reported to play an vital role in malignant cell mobility.^34^, ^35^ Recent studies showed ANXA2 could mediate upregulation of NF‐κB.^26^, ^27^ It is possible that S100A11 may promote GBM progression via regulating ANXA2‐mediated NF‐κB pathway. Here, we demonstrated that S100A11 knockdown reduced the levels of p‐Ikkα and p‐p65, and increased the IκBα. Additionally, p65 expression levels were induced in cytoplasmic and decreased in nuclear of knockdown S100A11 cells compared to control cells. Overexpression S100A11 had the opposite effects. Our results further revealed that the overexpression ANXA2 in S100A11‐silenced GBM cells can effectively rescue NF‐κB signalling pathway activation. More importantly, NF‐κB ‐p65 could regulate S100A11 at transcriptional level as a positive feedback. Therefore, the increased expression of S100A11 in GBM cells could promote GBM cell proliferation, EMT, migration, invasion and neurosphere formation by activating the ANXA2‐mediated NF‐κB signalling pathway.

In summary, our results demonstrate that S100A11 plays critical role in proliferation, EMT, migration, invasion and neurosphere formation in GBM cells and associated with poor survival of GBM patients. Moreover, functional and mechanistic studies suggest that S100A11/ANXA2/NF‐κB positive feedback loop in GBM cells that promote the progression of GBM. S100A11 may be a potential target for the development of antitumour drugs.

## CONFLICT OF INTEREST

The authors declare that they have no competing interests.

## AUTHOR CONTRIBUTION

JJ and TYM designed the experiments. TYM, XP and DXL performed the experiments. BZY, FL, SGC, ZPZ, CHL and LC analysed and interpreted the data. TYM was the major contributors in writing the manuscript.

## Data Availability

All the data and materials generated and/or analysed during the current study are available.
