# Supplementary material for: S100A11 functions as novel oncogene in glioblastoma via S100A11/ANXA2/NF‐κB positive feedback loop
Source: J Cell Mol Med. 2019 Aug 20;23(10):6907–18. doi: 10.1111/jcmm.14574 (PMC6787445; doi:10.1111/jcmm.14574)
Supplement: Supplementary file 1 [file JCMM-23-6907-s001.docx]

**Supplemental data**

**
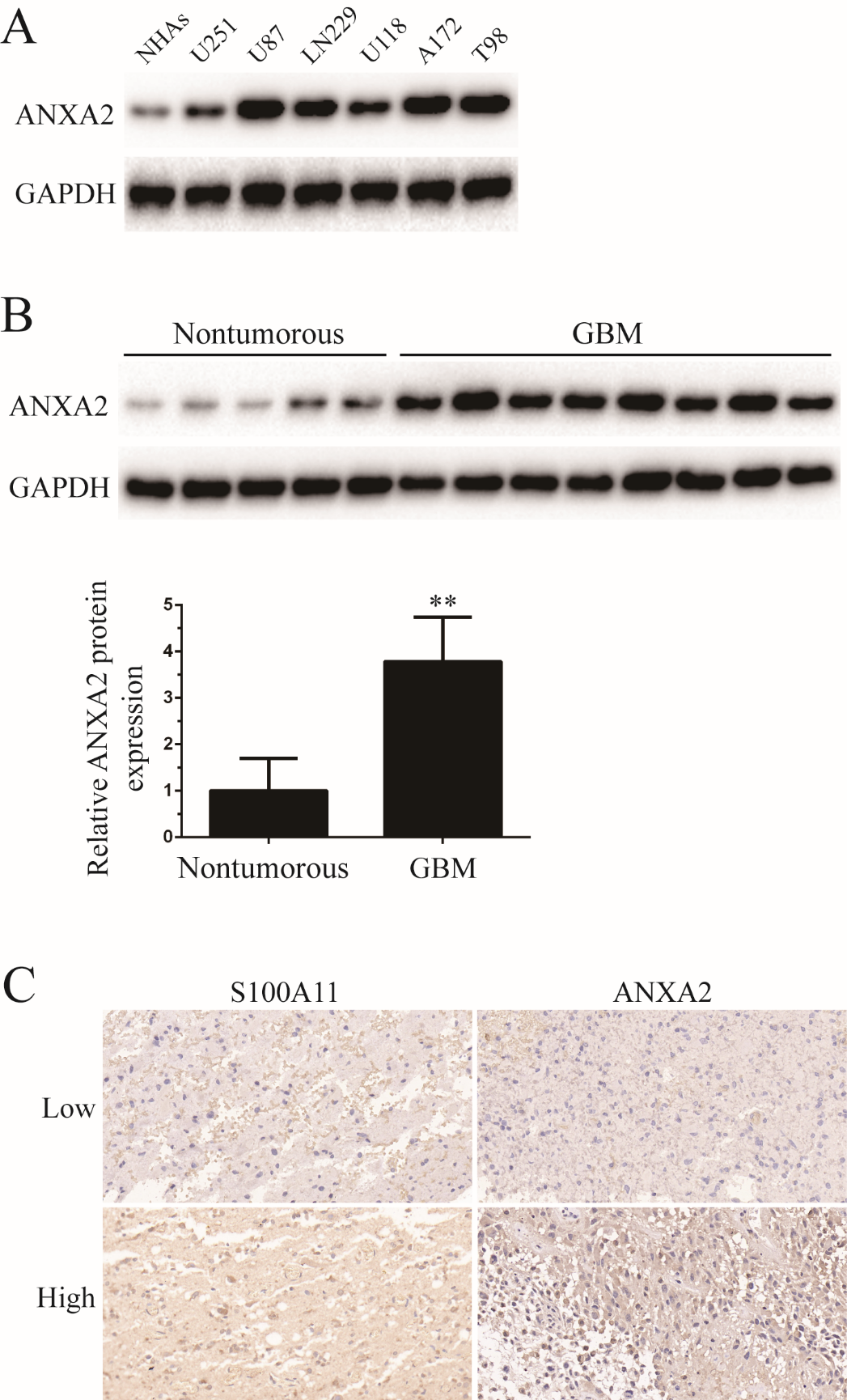
**

**Supplemental Fig S1. ANXA2 is upregulated in GBM cells and tissues, and S100A11 is correlated with ANXA2 protein expression. (A)** Western blot analysis of ANXA2 expression in NHAs and GBM cells. GAPDH was used as the loading control**. (B)** Western blot analysis of S00A11 expression in nontumorous brain tissues and GBM tissues (***P*<0.01). GAPDH was used as the loading control**.** Results are mean ± s.e.m. from at least 3 independent assays. **(C)** IHC staining of twenty human GBM specimens for S100A11 and ANXA2. Representative consecutive sections from 2 specimens are shown.
